# Supplementary material for: The Aspergillus flavus hacA Gene in the Unfolded Protein Response Pathway Is a Candidate Target for Host-Induced Gene Silencing
Source: J Fungi (Basel). 2024 Oct 16;10(10):719. doi: 10.3390/jof10100719 (PMC11508391; doi:10.3390/jof10100719)
Supplement: Supplementary file 1 [file jof-10-00719-s001.zip › jof-3207947-supplementary.pdf]

Table S1. Primers for construction and verification of integration of the *hacA* RNAi vectors

Primer	Sequence (5'→3')
StemF_P	TATCTGCAGGGAAGAGAAGAAGCCTGCAA
StemR_K	ATAGGTACCGGAAGAGAAGAAGCCTGCAA
LoopR	GCCACTCTTCACTCACAGACAACCGCTGAAGGAGGAAT
LoopF	GGGATGAAAGAAACGAATGACTAACAATAGATATAGAGTGCGGCTCGGGTGACACC
StmLp_BgII	ATTAGATCTTTCATCCCGCCACTCTTCACTCACAGACAACCGCTGAAGGAGGAA
LpStm_BgII	ATAAGATCTAACGAATGACTAACAATAGATATAGAGACAACCGCTGAAGGAGGA
CkRloop	GCCACTCTTCACTCACAGAC

TATAAGCTTTTCTCTTTAGAATTCAACTGTGGGTTTTGCTTTTTGCTTCATTCTCTTTGTCTTCT
CCATCTTTGATCAAATCCTGGACTTTCTCAATCCCCAGCTAATTCAATCATAGTCAGTTTTCTAT
TTTTATTATTTCTTTTTCTTTTGAAATGTGATTAACAACCAGTCTGTTATATATCTTGTACCCAG
ATTACGCCCAACTCGTGCTCCTCAGCCACAAAGATACTCAATTGATAGCCAAGATACATACATAC
CACAAAGTAAGGACTCCATGCATTGAGTATTACTCATCGTATTCTAGACTACTCCAAAACTCAGC
ACATAGACAAACAATACGAACCTCGTCTAAGGGTGATTCAGAGGCGGCAAAGCGGGGTTTTCGCA
TTTGATGTTCCTGGCACTTATGTAAGCCCACGCTTCCCGCTCAACTAAACCATCAGCCAATCAGA
CTGCTCAGATTTATCTTTTGAAGGGTATATAAATCATTGTAAAGAAGAACAAGT**CTGCAGTATCCC**
GGGTATGGTACCTTTTTTTTTGAGCATTTATCAGCTTGATATAGAGGTAGGAATGTATGGAGGTGC
AGAATGGCTATTTTGTTATTGGAGCGGGTTCGAAACGGAGGGCAGGAGACTTTTTTTAAATATGT

Figure S1. *A. flavus* U6 expression cassette cloned into a shortened pPTRII, from which the 3.0-kb *PstI* fragment was removed. The three restriction sites, *Pst*1-*Sma*I-*Kpn*I, are located between the U6 promoter and the U6 terminator. The promoter sequence is highlighted in blue, and the terminator sequence is highlighted in green.

A.

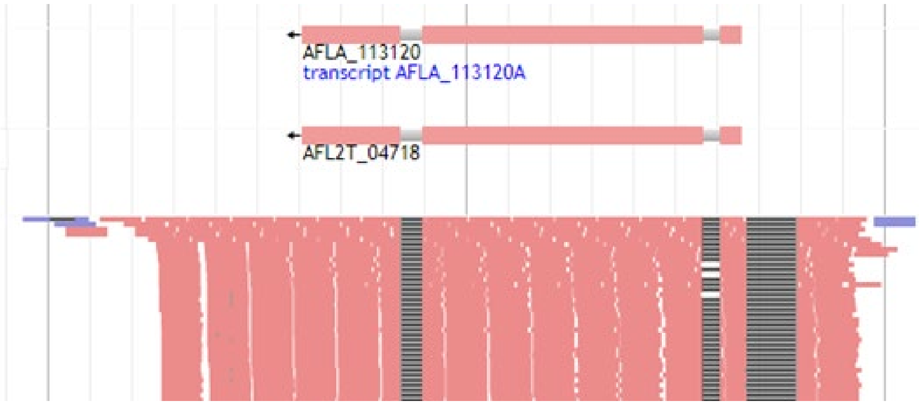

B.

ATGTTCATTAAATACGCTCTTCCCGCCCTGGCCGCGGCTCAGGCCGTCTTTGCTGCTAGTGATA**G**
TGAGTGAAGAGTGGCGCGATGAAAGAAACGAATGACTAACAATAGATAT**AG**AGTGCGGCTCGGGT
GACACCATCAAGATCGAGAACCAGAGCGATGCCGACGGCTACTCCAGCTGCAGCACCCTTAAGGG
TGATGTTGAGATCTCCGGAACCTACTCTGGCGATCTCCAACTCAATGGAGTTAAGCAGATCTCCG
GTGGACTGAGCTGCGATGGT

Figure S2. (A) Structure of *A. flavus* GPI (glycosylphosphatidylinositol-anchored protein) encoding gene, AFLA_007258 (=AFLA_113120). (B) Portion of the genomic sequence of the annotated *A. flavus* GPI gene showing the 52-bp intron, which was used as the loop portion for the RNAi construct. This intron is confirmed by RNA-seq data as the second intron. The yellow-highlighted GCG was changed to GGG to create a *Sma*I (-CCCGGG-) half site.

A.

HacA protein | length=438
MgSCDMEKTMSSVDSLPATPASEVPVLTVSPADTSLNSADVKTQEVKPEEKKPAKKRKSWGQELPVPKTNLPPRKRAKT
EDEKEQRRIERVLRNRAAAQTSRERKRLEMEKLENEKIQMEQQNQFLLQRLSQMEAENNRLSQQLAQLAAEVRGSRANT
PMPGSPATASPTLTPTLFKQERDELPLERIPFPTPSLSDYSPTLKPSTLAESSDVAQHPAAVLCDLQCPSLDSKEMEAP
SHFSTSAQTLNITLQMTLQLLFLTMTSTAYSTVIHPLNQILLSLKTGLPLMFSKEEIYQHFHLILWLISTPSLSPSKAS
RWPTGFRMRLLARLLACNPALARPLRDATGRALQLAVSENFSQGSMSVTDTQRSRWSWESLLTLSWAIDRLENPRRRRR
ILHGLRTSQIDRRNNLGKRQRSIRSTWSSNNTETLTSPLTGKDC

HacA$^{\Delta}$ protein | length=345
MSCDMEKTMSSVDSLPATPASEVPVLTVSPADTSLNSADVKTQEVKPEEKKPAKKRKSWGQELPVPKTNLPPRKRAKTE
DEKEQRRIERVLRNRAAAQTSRERKRLEMEKLENEKIQMEQQNQFLLQRLSQMEAENNRLSQQLAQLAAEVRGSRANTP
MPGSPATASPTLTPTLFKQERDELPLERIPFPTPSLSDYSPTLKPSTLAESSDVAQHPAVSVAGLEGDGSALPLFDLGS
DLKHHSTDDVAAPLSDDDFNRLFHGDSSVEPDSSVFEDGLAFDVLEGGDLSAFPFDSMVNFDSEPVTLEGIEMAHGLPD
ETTCKTSSVQPGFGASTTRCDGQGIAAGC

B. CRISPR site mutagenesis of the basic/acidic amino acid-coding region of *A. flavus hacA*

```
121         AAGACGCAGGAAGTCAAGCCGGAAGAGAAGAAGCCTGCAAAGAAGCGGAAGTCGTGGGGC  Bsc1
41           K  T  Q  E  V  K  P  E  E  K  K  P  A  K  K  R  K  S  W  G
181         CAAGAACTACCAGTTCCCAAGACCAACTTGCCTCCGAGGAAACGTGCCAAAACAGAAGAT  Bsc2/3
61           Q  E  L  P  V  P  K  T  N  L  P  P  R  K  R  A  K  T  E  D
241         GAGAAGGAGCAGCGTCGCATTGAACGCGTCCTTCGAAATCGTGCGGCAGCTCAGACGTCC
81           E  K  E  Q  R  R  I  E  R  V  L  R  N  R  A  A  A  Q  T  S
```

C. CRISPR site mutagenesis of the coding region of the leucine ZIP domain of *A. flavus hacA*

```
241         GAGAAGGAGCAGCGTCGCATTGAACGCGTCCTTCGAAATCGTGCGGCAGCTCAGACGTCC Zip1
81           E  K  E  Q  R  R  I  E  R  V  L  R  N  R  A  A  A  Q  T  S
301         CGGGAGCGCAAAAGGCTGGAAATGGAGAAGCTGGAGAATGAAAAAATTCAGATGGAACAA Zip2
101          R  E  R  K  R  L  E  M  E  K  L  E  N  E  K  I  Q  M  E  Q
361         CAGAATCAATTCCTCCTTCAGCGGTTGTCTCAGATGGAAGCAGAGAACAATCGCTTGAGC Zip3
121          Q  N  Q  F  L  L  Q  R  L  S  Q  M  E  A  E  N  N  R  L  S
421         CAACAGCTTGCTCAACTGGCTGCAGAGGTCCGCGGATCTCGTGCCAACACTCCAATGCCT
141          Q  Q  L  A  Q  L  A  A  E  V  R  G  S  R  A  N  T  P  M  P
```

D. CRISPR site mutagenesis of the non-conventional intron (Nt20) of *A. flavus hacA*

```
601         TTGAAGCCTTCCACTCTGGCTGAGTCCTCCGACGTGGCACAACATCCTGCAGCGGTGTTG Nt20
201          L  K  P  S  T  L  A  E  S  S  D  V  A  Q  H  P  A  A  V  L
201          L  K  P  S  T  L  A  E  S  S  D  V  A  Q  H  P   (HacAΔ)
661         TGTGACCTGCAGTGTCCGTCGCTGGACTCGAAGGAGATGGAAGCGCCCTCCCACTTTTCG
221          C  D  L  Q  C  P  S  L  D  S  K  E  M  E  A  P  S  H  F  S
217                A  V  S  V  A  G  L  E  G  D  G  S  A  L  P  L  F  D
```

Figure S3. Two forms of HacA (A) and CRISPR/Cas9 target sites in *A. flavus hacA* gene (B, C, and D). Underlined, yellow-highlighted nucleotides indicate protospacer sequences used to generate sgRNAs targeting selected regions of the *hacA* gene. The protospacer adjacent motifs (PAMs i.e., NGG) are shown in bold black. Basic amino acids, K and R, are highlighted in bold red, while acidic amino acids, D and E, are highlighted in bold green. The nine leucines (L) in the bZip domain are highlighted in bold blue. The 20-nucleotide intron, which is removed in the mature *hacA* transcript under ER stress conditions, is double underlined, and the target region is highlighted in grey.

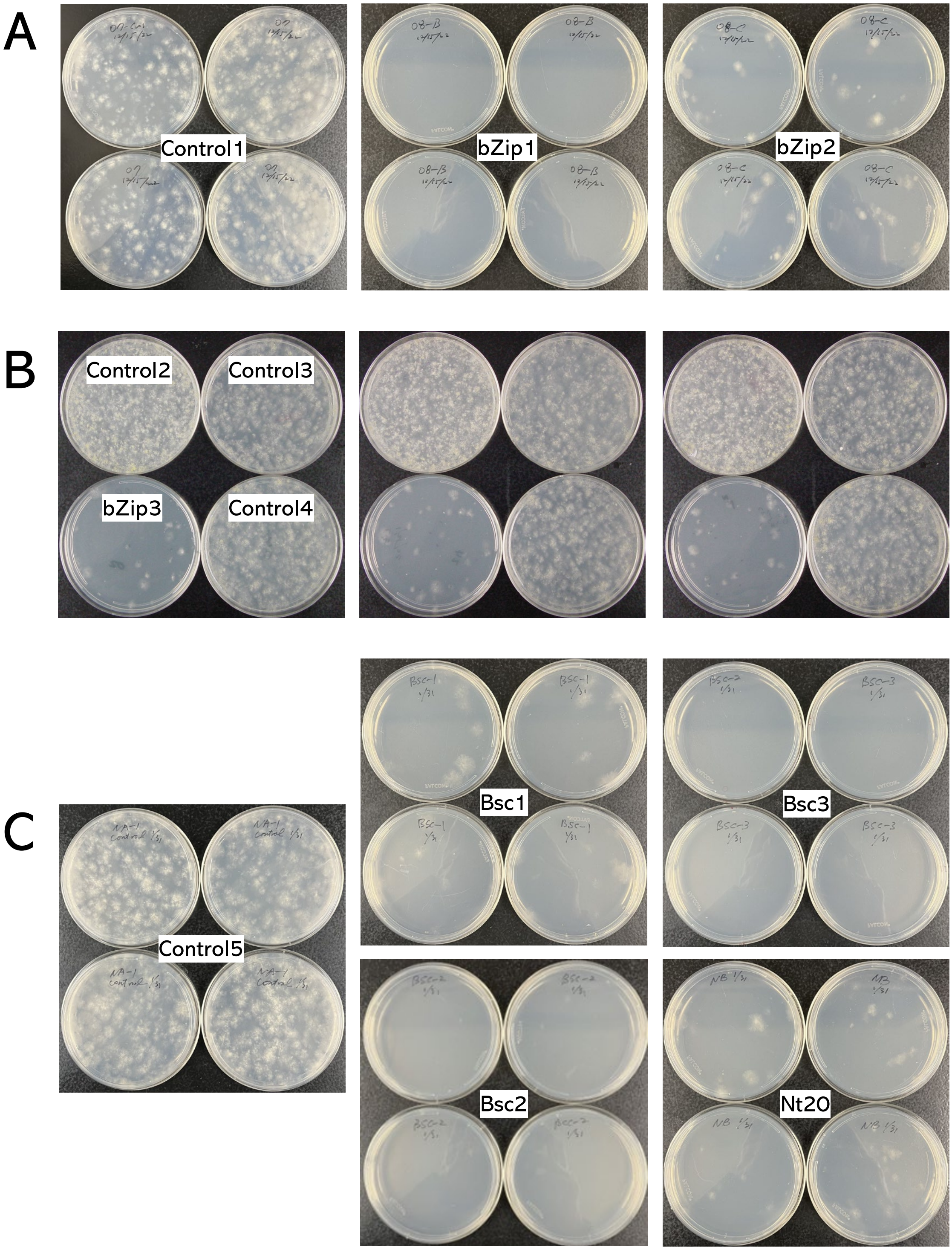

Figure S4. Primary transformants on CZ regeneration plates. Transformation experiments in each panel (A, B, and C) were performed simultaneously. Approximately 2.0×10^6 protoplasts and 250 ng of a CRISPR/Cas9 vector were used in each transformation. Three or four of the six agar plates from each set are shown. The plates were incubated at 30°C for four days.

A (I) (II)

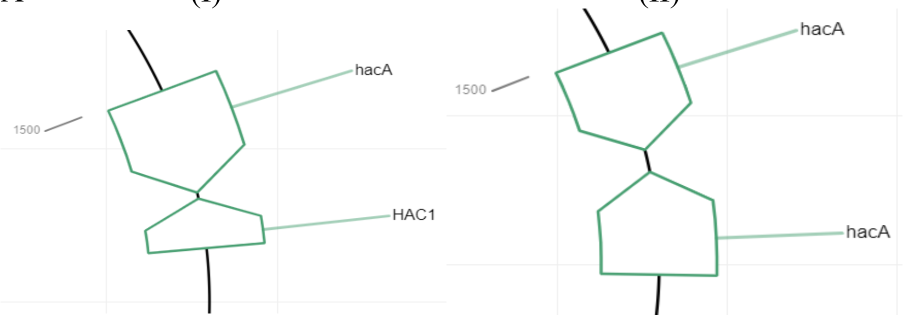

B (I)

```
1    GGAAGAGAAGAAGCCTGCAAAGAAGCGGAAGTCGTGGGGCCAAGAACTACCAGTTCCCAA  60
     ||||||||||||||||||||||||||||||||||||||||||||||||||||||||||||
1    GGAAGAGAAGAAGCCTGCAAAGAAGCGGAAGTCGTGGGGCCAAGAACTACCAGTTCCCAA  60
61   GACCAACTTGCCTCCGAGGAAACGTGCCAAAACAGAAGATGAGAAGGAGCAGCGTCGCAT  120
     ||||||||||||||||||||||||||||||||||||||||||||||||||||||||||||
61   GACCAACTTGCCTCCGAGGAAACGTGCCAAAACAGAAGATGAGAAGGAGCAGCGTCGCAT  120

121  TGAACGCGTCCTTCGAAATCGTGCGGCAGCTCAGACGTCCCGGGAGCGCAAAAGGCTGGA  180
     ||||||||||||||||||||||||||||||||||||||||||||||||||||||||||||
121  TGAACGCGTCCTTCGAAATCGTGCGGCAGCTCAGACGTCCCGGGAGCGCAAAAGGCTGGA  180
181  AATGGAGAAGCTGGAGAATGAAAAAATTCAGATGGAACAACAGAATCAATTCCTCCTTCA  240
     ||||||||||||||||||||||||||||||||||||||||||||||||||||||||||||
181  AATGGAGAAGCTGGAGAATGAAAAAATTCAGATGGAACAACAGAATCAATTCCTCCTTCA  240
241  GCGGTTGTCTGTGAGTGAAGAGTGGCGGGATGAAAGAAACGAATGACTAACAATAGATAT  300
     |||||||||||||||||||||||||
241  GCGGTTGTCTGTGAGTGAAGAGTGG-----------------------------------  270
301  AGAGACAACCGCTGAAGGAGGAATTGATTCTGTTGTTCCATCTGAATTTTTTCATTCTCC  360
     ------------------------------------------------------------
361  AGCTTCTCCATTTCCAGCCTTTTGCGCTCCCGGGACGTCTGAGCTGCCGCACGATTTCGA  420
                                   ||||||||||||||||||||||||||||||
271  ------------------------------CGGGACGTCTGAGCTGCCGCACGATTTCGA  295
421  AGGACGCGTTCAATGCGACGCTGCTCCTTCTCATCTTCTGTTTTGGCACGTTTCCTCGGA  480
     ||||||||||||||||||||||||||||||||||||||||||||||||||||||||||||
296  AGGACGCGTTCAATGCGACGCTGCTCCTTCTCATCTTCTGTTTTGGCACGTTTCCTCGGA  355
481  GGCAAGTTGGTCTTGGGAACTGGTAGTTCTTGGCCCCACGACTTCCGCTTCTTTGCAGGC  540
     ||||||||||||||||||||||||||||||||||||||||||||||||||||||||||||
356  GGCAAGTTGGTCTTGGGAACTGGTAGTTCTTGGCCCCACGACTTCCGCTTCTTTGCAGGC  415
541  TTCTTCTCTTCC---------------  552
     ||||||||||||
416  TTCTTCTCTTCCGGTACCTTTTTTTTT  442
```

B (II)

```
1     GGAAGAGAAGAAGCCTGCAAAGAAGCGGAAGTCGTGGGGCCAAGAACTACCAGTTCCCAA
61    GACCAACTTGCCTCCGAGGAAACGTGCCAAAACAGAAGATGAGAAGGAGCAGCGTCGCAT
121   TGAACGCGTCCTTCGAAATCGTGCGGCAGCTCAGACGTCCCGGGAGCGCAAAAGGCTGGA
181   AATGGAGAAGCTGGAGAATGAAAAAATTCAGATGGAACAACAGAATCAATTCCTCCTTCA
241   GCGGTTGTCTGTGAGTGAAGAGTGGCGGGATGAAAGATCTAACGAATGACTAACAATAGA
301   TATAGAGACAACCGCTGAAGGAGGAATTGATTCTGTTGTTCCATCTGAATTTTTTCATTC
361   TCCAGCTTCTCCATTTCCAGCCTTTTGCGCTCCCGGGACGTCTGAGCTGCCGCACGATTT
421   CGAAGGACGCGTTCAATGCGACGCTGCTCCTTCTCATCTTCTGTTTTGGCACGTTTCCTC
481   GGAGGCAAGTTGGTCTTGGGAACTGGTAGTTCTTGGCCCCACGACTTCCGCTTCTTTGCA
541   GGCTTCTTCTCTTCCGGTACCTTTTTTTTT
```

Figure S5. Graphic representations and sequences of the stem and loop portions of two *hacA* RNAi constructs. (A) The stem of construct I is 162 bps, and the stem of construct II is 250 bps. (B) The *Sma*I site, CCCGGG, in the stem seqeuence resulting in a deletion in the first *hac*A RNAi construct is shown in bold red (I). Color coded portions of the stem (green and blue) and the loop (yellow) sequences are indicated. The consensus splicing sites, GT and AG, in the modifed GPI gene intron are shown in bold red. GGTACC is the tagged *Kpn*I site before the U6 termination sequence (I and II).

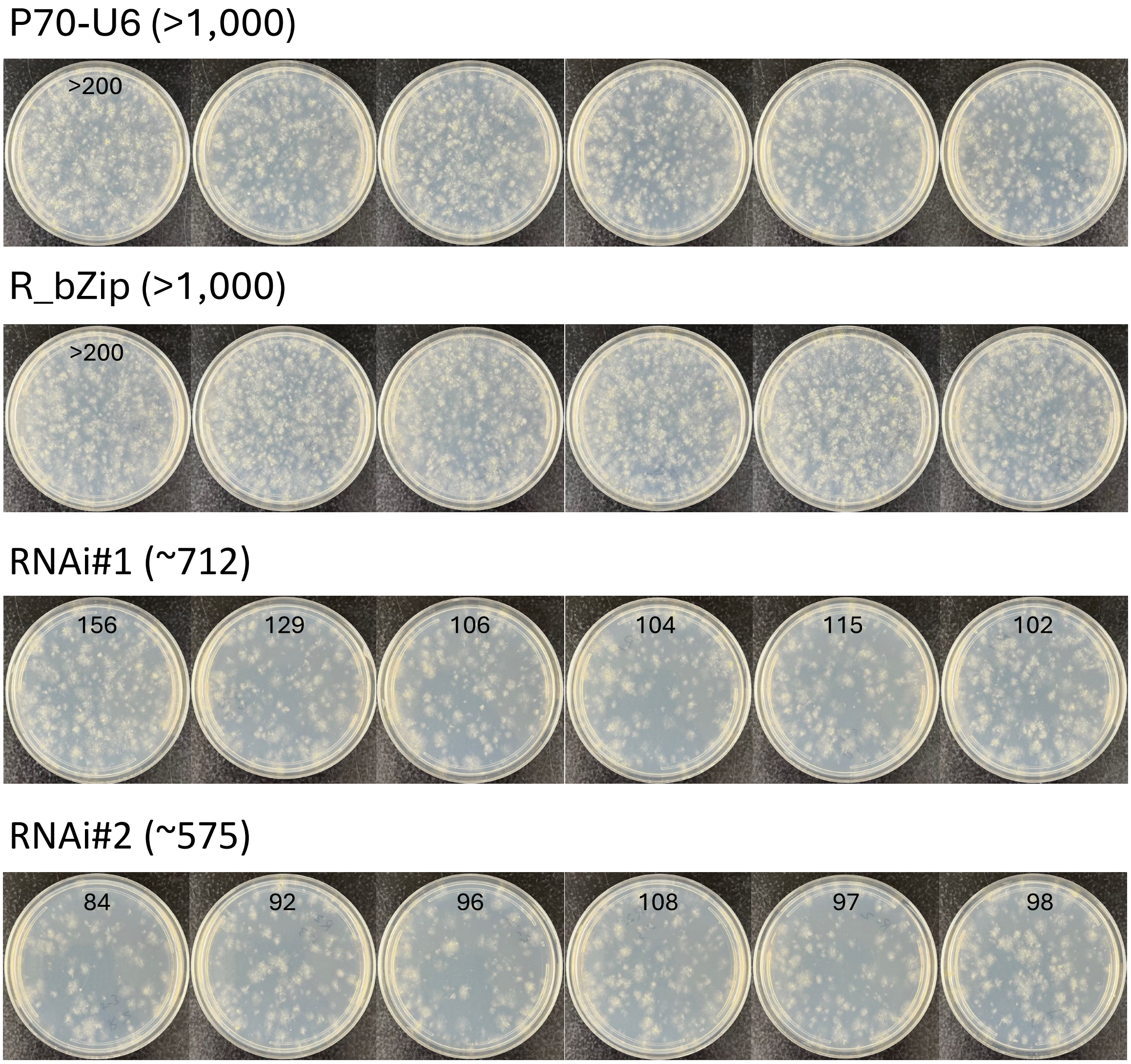

Figure S6A. Primary transformants of *A. flavus* CA14 on CZ regeneration plates. The two controls are the P70-U6 cloning vector and the R_bZip half stem-loop structure construct. Two independent *hacA* RNAi constructs were tested. Approximately 1.0 µg *Hind*III linearized DNA and 2.0×10^6 protoplasts were used in each transformation. Cultures were incubated at 30°C for four days in the dark.

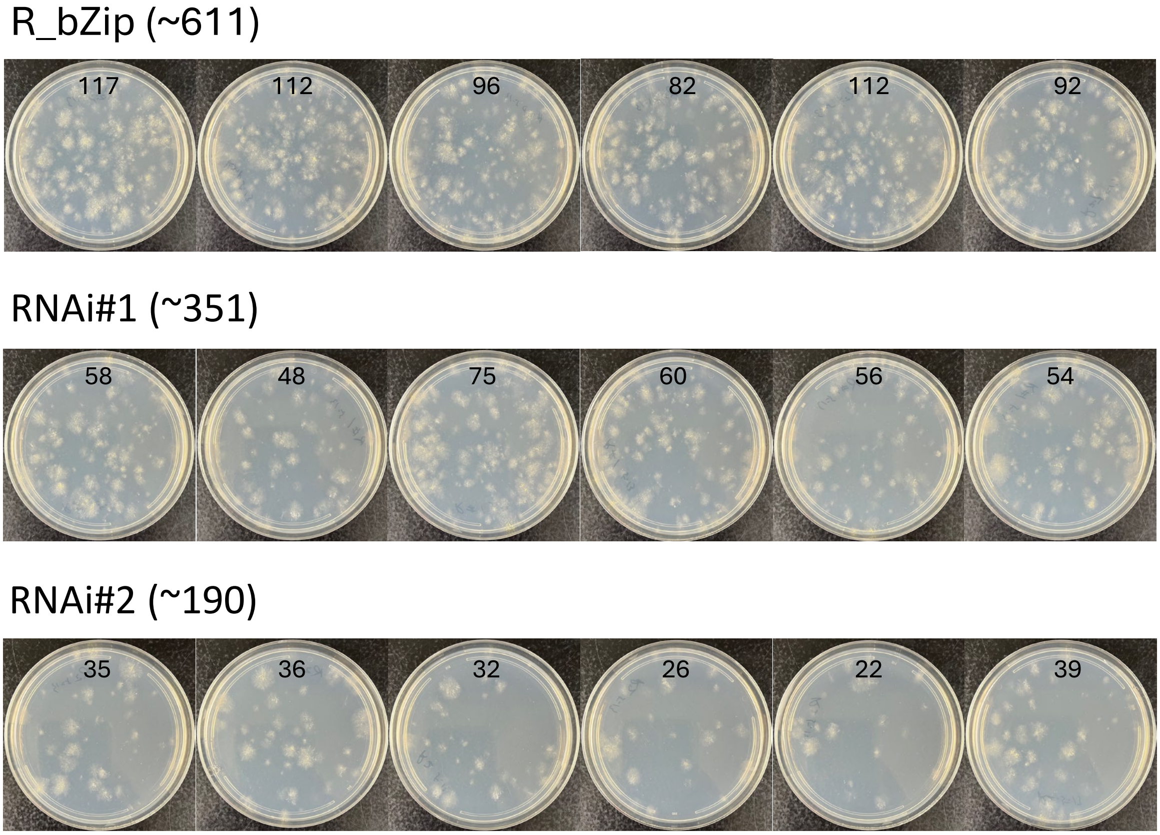

Figure S6B. Primary transformants of *A. flavus* CA14 on CZ regeneration plates. The control was the R_bZip construct containing a half stem-loop structure. Two independent *hacA* RNAi constructs were tested. Approximately 1.0 µg *Hind*III linearized DNA and 1.0×10^6 protoplasts were used in each transformation. Cultures were incubated at 30°C for four days in the dark.

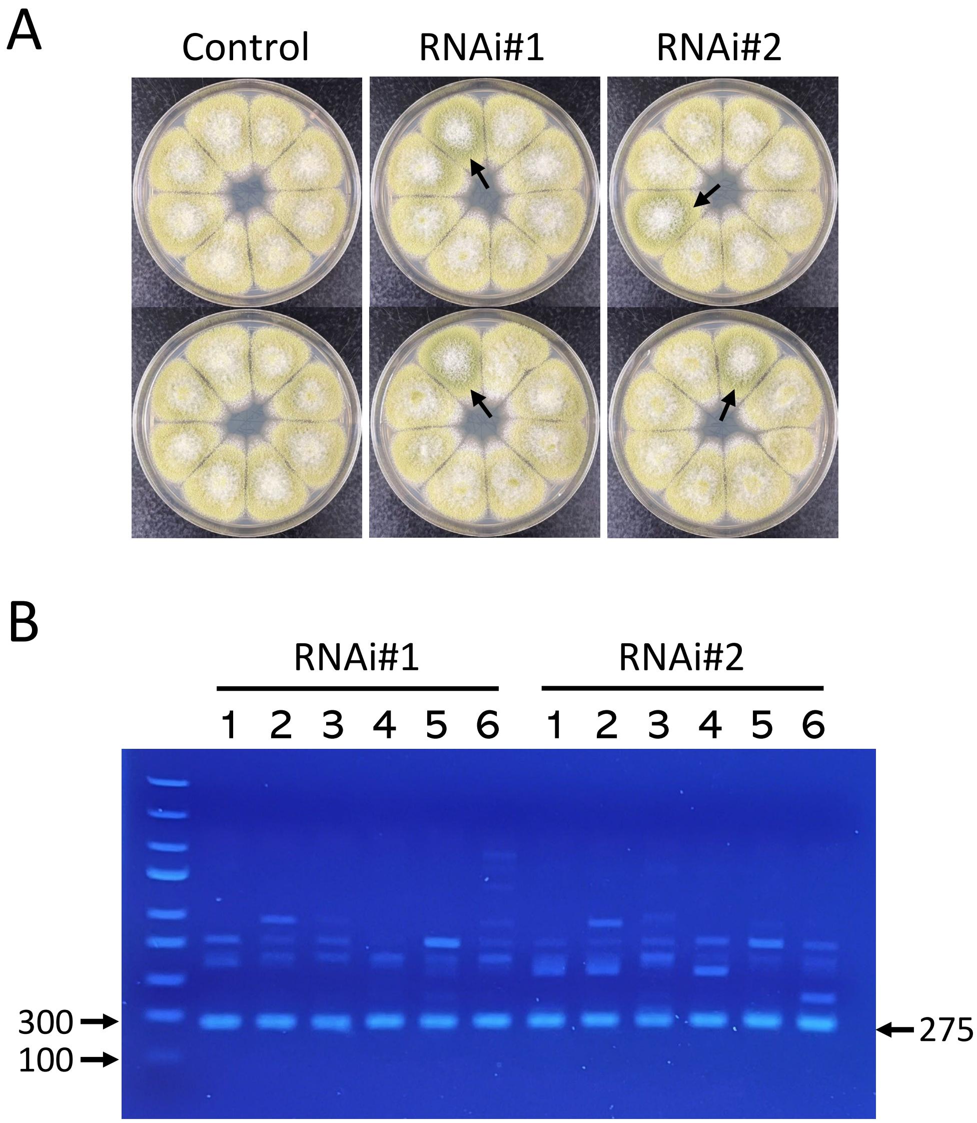

Figure S7. (A) Colony morphology of *hacA* RNAi transformants on PDA plates. Transformants exhibiting intense green, velvet-like colony morphology are indicated by arrows. (B) PCR results show that the *hacA* RNAi cassette was integrated into the genome of *hacA* RNAi transformants. The primers StemF_P and CkRloop (Table S1), amplifying the left-half part of the loop-stem structure of the RNAi construct, were used in direct colony PCR,.
